# Carcinogenicity of Aspartame in Rats Not Proven

**DOI:** 10.1289/ehp.10881

**Published:** 2008-06

**Authors:** Bernadene Magnuson, Gary M. Williams

**Affiliations:** Department of Nutrition and Food Science, University of Maryland, College Park, Maryland, E-mail: bmagnuso@umd.edu; Department of Pathology, New York Medical College, Valhalla, New York

In their article on lifetime exposure to aspartame in rats, Soffritti et al. (2007) purported that their study demonstrated increased carcinogenic effects in female rats as a result of exposure beginning during prenatal life.

We believe that this article (Soffritti et al. 2007) has methodologic and conceptual weaknesses that require exposition. First, although the study was a toxicology study, the most important element—the reported doses—are not correct. The doses are “estimates” based on assuming constant food consumption of 20 g/day and constant body weights of 400 g for each rat from *in utero* (fetal day 12) to death. These assumptions are unrealistic and inaccurate. The doses during the early growth phase of rats would be much higher because, as is well known, rats consume more food per gram of body weight during the rapid growth phase. Food consumption and body weight were reportedly measured throughout the experiment; however, Soffritti et al. (2007) presented only data beginning 16 weeks postpartum, when rats reached adult body weight. Therefore the authors’ conclusions are built on the exposure period for which they provide no data.

Second, for a study allegedly designed to assess prenatal exposure, Soffritti et al. (2007) did not address important details, such as *a*) pregnancy history and ages of breeders; *b*) number of pregnant dams per dose group; *c*) growth and food consumption of mothers during pregnancy and lactation; *d*) pregnancy outcomes; *e*) disposition of pups from all mothers and each litter; *f*) the origin of the 70 pups; and *g*) body weight of pups at birth and during lactation. These details are typically required to allow other scientists to assess the appropriateness of the study design and to repeat the study, if desired.

The findings are of questionable biological significance for a number of reasons. The lymphoma/leukemia incidences in the high-dose group, which were the only significant differences from control, were within or near the reported historical control ranges. Similarly, the mammary gland carcinoma incidence in high-dose females (again, the only significant difference from control) was similar to historical controls. In their article, Soffritti et al. (2007) stated that their study disproved the conclusions of the European Food Safety Authority (EFSA 2006) that the incidences of lymphomas/leukemias observed in the first report (Soffritti et al. 2006) were “unrelated to aspartame given the high background incidence of chronic inflammatory changes in the lungs …” (EFSA 2006). The U.S. Food and Drug Administration (FDA 2007) agreed with the EFSA assessment. It is not clear to us how this study disproved the EFSA’s conclusions. Soffritti et al. (2007) indicated that the lung was often the site of lymphoma again in this study, which is not surprising because they used the same infected colony. Studies in the 1960s demonstrated that the progression of chronic pneumonia in rats resulted in lymphoid neoplasmas, and elimination of chronic respiratory disease in rat colonies reduced the incidence of pulmonary lymphoid neoplasias to near zero (Cotchin and Roe 1967). Rats with pulmonary infections developed lesions in multiple sites earlier than rats free from pulmonary disease (Cotchin and Roe 1967). The establishment of pathogen-free animal suppliers for toxicity research was impelled for this reason. Therefore, we believe it is highly likely that the present findings are due to infection and not aspartame consumption.

Data do not support the conclusions of Soffritti et al. (2007) that aspartame has carcinogenic potential at doses near the human level of exposure. The authors observed no significant effects at the low-diet level, and the actual dose is unknown. Also, no data were provided on *in utero* exposure. Aspartame is completely digested in the gastrointestinal tract into two amino acids (phenylalanine and aspartic acid) and methanol, which is subsequently metabolized to carbon dioxide and water. In human clinical studies (reviewed by Stegink and Filer 1996), oral doses equal to or exceeding the amount that would represent the 99th percentile of aspartame intake did not increase plasma aspartate or phenylalanine levels in adults or children, or in breast milk from lactating women beyond normal postprandial concentrations. Ratios of fetal/maternal plasma amino acids and transport across the placental membrane were unchanged in pregnant rabbits that received 1,600 mg aspartame/kg/day (Ranney et al. 1975). Thus, a biologically plausible explanation is lacking for Soffritti et al.’s (2007) contention that prenatal exposure to aspartame increases cancer risk.

In summary, considering that there are no significant differences in cancer rates between high-dose groups and historical controls, plus the many deficiencies in the experimental design and data, Soffritti et al. (2007) failed to provide convincing evidence of aspartame carcinogenicity. Given the effort expended by many government review agencies to document shortcomings of the first article by this group (Soffritti et al. 2006), it is disappointing that the editor and reviewers of this paper (Soffritti et al. 2007) did not require the authors to address those problems that appear again in this study. Diligence is especially necessary on topics of great public interest and relevance because the public is relying upon the scientific community to assure that only high quality, well-documented, and controlled studies appear in peer-reviewed journals.
